# A Genome-Wide Association Study Identifies Two Novel Promising Candidate Genes Affecting *Escherichia coli* F4ab/F4ac Susceptibility in Swine

**DOI:** 10.1371/journal.pone.0032127

**Published:** 2012-03-23

**Authors:** Wei-Xuan Fu, Yang Liu, Xin Lu, Xiao-Yan Niu, Xiang-Dong Ding, Jian-Feng Liu, Qin Zhang

**Affiliations:** 1 Key Laboratory of Animal Genetics Breeding and Reproduction, Ministry of Agriculture, College of Animal Science and Technology, China Agricultural University, Beijing, China; 2 State Key Laboratory for Infectious Disease Prevention and Control, National Institute for Communicable Disease Control and Prevention, Chinese Centre for Disease Control and Prevention, Beijing, China; American University in Cairo, Egypt

## Abstract

Enterotoxigenic *Escherichia coli* (ETEC) expressing F4 fimbria is the major pathogenic bacteria causing diarrhoea in neonatal and post-weaning piglets. Previous studies have revealed that the susceptibility to ETEC F4ab/F4ac is an autosomal Mendelian dominant trait and the loci controlling the F4ab/F4ac receptor are located on SSC13q41, between markers *SW207* and *S0283*. To pinpoint these loci and further validate previous findings, we performed a genome-wide association study (GWAS) using a two generation family-based population, consisting of 301 piglets with phenotypes of susceptibility to ETEC F4ab/F4ac by the *vitro* adhesion test. The DNA of all piglets and their parents was genotyped using the Illumina PorcineSNP60 BeadChip, and 50,972 and 50,483 SNPs were available for F4ab and F4ac susceptibility, respectively, in the association analysis after quality control. In summary, 28 and 18 significant SNPs (*p*<0.05) were detected associated with F4ab and F4ac susceptibility respectively at genome-wide significance level. From these significant findings, two novel candidate genes, *HEG1* and *ITGB5*, were firstly identified as the most promising genes underlying F4ab/F4ac susceptibility in swine according to their functions and positions. Our findings herein provide a novel evidence for unravelling genetic mechanism of diarrhoea risk in piglets.

## Introduction

Susceptibility to enterotoxigenic *Escherichia* (*E.) coli* (ETEC) with F4 (K88) fimbriae is dominantly inherited in neonatal and pre-weaning piglets, potentially causing the diarrhoea and death of piglets. Antigenically, three fimbriae variants have been identified, i.e., F4ab, F4ac and F4ad [1]. Diarrhoea due to ETEC F4 (mainly F4ab and F4ac) infection is very common over the world where pigs are raised in large numbers [2]. Once the bacteria adhere to the brush border of enterocytes and colonize the small intestine, the enterotoxins produced by them induce an increased secretion of electrolytes into lumen, resulting in diarrhoea. However, not all piglets are susceptible to ETEC F4 and the susceptibility is determined by the existence of a specific receptor of ETEC F4ab/F4ac [3].

Identification of causal mutation(s) affecting F4ab/F4ac susceptibility is a feasible way for controlling piglet diarrhoea. In the past few years, several groups have focused on mapping potential genes of F4ab/F4ac receptor (*F4abR/F4acR*), which has been mapped on SSC13 initially [4]. Python *et al.* refined the localization of the *F4acR* gene in the interval of *S0068* and *SW1030*, close to *S0075* and *SW225* [5], and four functional genes in the region between *SW207* and *S0283* were considered as candidates of *F4acR* in their further research, but no mutations were found in the cDNA sequences of these genes associated with the *F4acR* genotypes [6]. A linkage and comparative mapping study demonstrated that the most likely region of *F4abR/F4acR* gene(s) was between *SW207* and *S0075* [7]. Studies [3], [8], [9] suggested that the *MUC4* (mucin 4) gene should be considered as one of the most promising candidate genes for F4abR/F4acR based on three aspects: (1) its physical position falls into the region identified in most of linkage analyses, (2) the protein encoded by the *MUC4* gene is one of mucin-like sialoglycoprotein which the ETEC F4 maybe adheres to, and (3) association studies showed strong association between a mutation within the gene and the adhesive phenotypes. However, in a recent study, Rampoldi *et al.* found that the region of *F4abR/F4acR* gene(s) was distal to the interval between the *MUC4* gene and the *LMLN* gene (leishmanolysin-like gene) through testing recombination events in three–generation pedigree [10]. Findings from these studies suggest that further endeavours are still expected to explore more convincing evidences on the *F4abR/F4acR* gene(s).

Although QTL mapping has been very successful in domestic animals for many complex quantitative traits and a few prominent findings have been reported [11], [12], [13], [14], identification of quantitative trait mutations (QTMs) is still a challenge due to the commonly existing limitations of linkage analysis [15]. Recently, the first high-density 60 K porcine SNP array has been developed [16], which offers the prerequisite of genome-wide association study (GWAS) in swine, a powerful approach for high-resolution mapping of loci controlling phenotypic traits. Duijvesteijn *et al.* [17] reported a GWAS for androstenone levels in pigs, which reveals a cluster of candidate genes on chromosome 6. Moreover, Pryce *et al.* [18] and Jiang *et al.* [19] performed GWAS to identify loci affecting milk production traits in dairy cattle in two different populations and obtained very similar results. More recently, Fan *et al.* [20] performed a GWAS for body composition and structural soundness traits of pigs and identified several genes by functional clustering analysis. Up to now, GWAS has acted as a most commonly used strategy for gene identification for complex traits in animals as well as humans.

Motivated by further clarifying genetic basis of ETEC F4ab/F4ac susceptibility and pursuing more confirmatory evidences of *F4abR/F4acR* gene(s), we performed a GWAS for high-resolution identification of loci controlling F4ab/F4acsusceptibility in swine using a case-control design. Our study identified 28 and 18 significant SNPs for ETEC F4ab/F4ac susceptibility, respectively. These significant findings indicate two genes, *HEG1* and *ITGB5*, can be treated as novel promising candidates underlying F4ab/F4ac susceptibility in swine according to their functions and positions.

## Materials and Methods

### Animal resource

The animal resource used for this study is a two generation family-based population, which is a subset of the population of our previous study [21], consisting of 301 pure bred piglets of three breeds, among which 67 were Landrace offspring of 4 boars and 13 sows, 161 were Yorkshire offspring of 7 boars and 29 sows, and 73 were Songliao Black (a Chinese native breed) offspring of 3 boars and 13 sows (see Table 1). The pigs were raised under standard indoor conditions at the experimental farm of the Institute of Animal Sciences, Chinese Academy of Agricultural Sciences. At 35 days of age, all piglets were slaughtered, and their jejunum and ear tissue samples were collected. The ear tissue samples of 69 parents recorded in the original pedigree were also collected. In total, there were 370 pigs in our study, including 301 piglets and 69 parental individuals.

**Table 1:** Family structure in three swine breeds.

Breed^a^	No. piglet	Original parents^b^		Corrected parents^c^	
		No. boars	No. sows	No. boars	No. sows
**LR**	67	4	13	6	16
**LW**	161	7	29	8	37
**SB**	73	3	13	3	13

a LR, Landrace; LW, Yorkshire; SB, Songliao Black;

b Parents in the original parentage records;

c Parents after correction for the original parentage errors using SNP genotype information.

The whole procedure for the collection of jejunum and ear tissue samples and the slaughter of piglets was carried out in strict accordance with the protocol approved by the Animal Welfare Committee of China Agricultural University (Permit Number: DK996).

### Measurement of phenotypes

All 301 piglets were phenotyped for ETEC F4ab/F4ac susceptibility using in *vitro* adhesion test. Two ETEC F4 strains (195 (F4ab, C83901, O8:K87) and 200 (F4ac, C83907, O149:K91)) and a bovine-origined *E. coli* strain (238 (C83286, O38:K99)) as negative control were provided by the China Institute of Veterinary Drug Control. The procedures of collecting jejunal epithelial cells, preparing bacterial suspension, in *vitro* adhesion test and classification of adhesion phenotypes (strongly adhesive, adhesive, weakly adhesive and non-adhesive) herein were described in detail in our previous report [21].

For the case-control design in our GWAS, the four phenotype categories were further classified into two classes. The non-adhesive and weakly adhesive phenotypes were classified to as negative (control) and the other two categories as positive (case). The distribution of the two classes in the three breeds is given in Table 2.

**Table 2:** Distribution of F4ab/F4ac adhesion phenotypes in three swine breeds.

	F4ab				F4ac			
Breed^a^	Total	LR	LW	SB	Total	LR	LW	SB
**Negative (Control)** b	140	11	74	55	168	16	85	67
**Positive (Case)** c	161	56	87	18	133	51	76	6
**Total**	301	67	161	73	301	67	161	73

a LR, Landrace; LW, Yorkshire; SB, Songliao Black;

b Including both non-adhesive and weakly adhesive piglets;

c Including both adhesive and strongly adhesive piglets.

### Genotyping and quality control

Extracted from ear tissue samples of all piglets and their parents, DNA was genotyped using the Illumina PorcineSNP60 BeadChip containing 62,163 SNPs. Features of the chip have been detailed previously [16]. The genotypes were judged using BeadStudio (Version 3.2.2, Illumina, lnc.) and a custom cluster file developed from the 370 samples.

To assess the technical reliability of the genotyping panel, a randomly selected DNA sample was genotyped twice, and over 99% identity of called genotypes was obtained. This demonstrates the technically robust feature of the 60 K SNP BeadChip panel employed herein.

Like most GWAS using the case-control design [22], [23], quality control procedures of the genotype data were performed as follows. First, only samples with a minimum of 90% call rate were included. Second, out of the initial full set of 62,163 SNPs, we discarded: (1) SNPs with a call rate <90% (n = 3,773) in all piglets; (2) those severely deviating from Hardy–Weinberg equilibrium (HWE) (*p*<10E-6) in the two control groups (n = 2,179 for F4ab and n = 2,693 for F4ac); and (3) those having a minor allele frequency (MAF)<0.03 in all piglets (n = 7,797). After quality control, 50,972 and 50,483 SNPs were available for F4ab and F4ac respectively in the subsequent analyses, and their distributions in the porcine genome are presented in Table 3.

**Table 3:** Distribution of SNPs on chromosomes after quality control and the average distances between adjacent SNPs.

Chr.	F4ab		F4ac	
	No. SNPs	Average distance (kb)^a^	No. SNPs	Average distance (kb)^a^
**1**	5,439	54.32	5,362	55.10
**2**	2,822	49.60	2,785	50.26
**3**	2,352	52.41	2,333	52.84
**4**	3,132	43.52	3,119	43.70
**5**	2,005	50.14	1,993	50.44
**6**	2,463	49.94	2,457	50.06
**7**	2,963	46.01	2,937	46.42
**8**	2,178	54.91	2,152	55.58
**9**	2,711	48.87	2,684	49.36
**10**	1,377	47.66	1,354	48.47
**11**	1,634	48.84	1,613	49.48
**12**	1,288	44.57	1,275	45.03
**13**	3,025	47.98	3,005	48.30
**14**	3,481	42.66	3,443	43.13
**15**	2,265	59.31	2,220	60.51
**16**	1,536	50.31	1,540	50.18
**17**	1,377	46.54	1,364	46.99
**18**	1,112	48.75	1,104	49.1
**X**	985	127.60	981	128.12
**0** b	6,827	NA	6,762	NA
**TOTAL**	50,972		50,483	

a Derived from the most recent porcine genome sequence assembly (Sscrofa9.2).

(http://www.ensembl.org/Sus_scrofa/Info/Index);

b These SNPs are not assigned to any chromosomes. NA: not available.

### Parentage test

Considering the probability of potential parentage mistakes in the original parentage records, we adopted Cervus (Version 3.0) [24] to infer the most possible parent-offspring pairs with maximum likelihood method using 200 randomly chosen autosomal SNPs with more than 99% call rate. Accordingly, among all 301 piglets, a total of 50 (16.6%) individuals had parentage errors in the original records, including 11 with incorrect paternal records, 31 with incorrect maternal records and 8 with both incorrect paternal and maternal records. Since the parentage information was to be used in the association analysis, parentage correction was further conducted. Among the 50 piglets with parentage errors, 9 were reassigned to the correct parents among the 69 known parents, while 41 were unable to be assigned to any of the known fathers or mothers. Assuming correct sibship information in the original parentage records, they were assigned to be offspring of 14 unknown parents including 3 boars and 11 sows. Hence, in our study, the 301 piglets were actually from 83 parents. The corrected parentage information was used in the subsequent analyses. The information of both original and corrected parentage is given in Table 1.

### Association Analysis

Compared with traditional population-based case-control design in GWAS, individuals in cases (piglets with adhesive phenotypes) and controls (piglets with non-adhesive phenotypes) in our studies are related within each breed, and heterogeneity also exists among three different breeds, which may potentially induce confounding in the analysis. To overcome this limitation, we used a recently published program ROADTRIPS (Version 1.2) [25] to perform the association analysis. An important advantage of ROADTRIP is that it can simultaneously deal with data with pedigree structure as well as population admixture in association test. In ROADTRIP, an empirical covariance matrix **Ψ** constructed using genome-wide SNP data is employed to adjust for potential population admixture as well as relatedness among individuals, while maintaining the advantage of utilizing known pedigree information when it is available.

ROADTRIPS provides three association tests named RM test, RW test and Rχ test, respectively. Compared with the RW and Rχ tests, the RM test can use the phenotypic information for individuals with missing genotypes provided they have a sampled relative who is genotyped at the tested marker. The RM and RW tests can improve power by using this information when partial or complete pedigree information is available. Furthermore, the RM test is the most powerful in a general class of linear statistics under the framework of two-allele disease model for outbred populations.

Considering features of the RM test aforementioned as well as the data structure of our study based on the corrected pedigree above, we adopted the test to detect loci associated with susceptibility to ETEC F4ab/F4ac, and the *p* values for the RM statistic were derived from an asymptotic chi-square distribution with 1 degree of freedom.

For the significant SNPs detected by the RM test, linkage disequilibrium (LD) patterns between them were quantified as *r^2^* using Haploview (Version 4.2) [26] and the LD blocks were defined by the criteria of Gabriel *et al.* [27].

### Statistical Inference

In the study, the permutation method was adopted to adjust for multiple testing for the number of SNPs tested through constructing a genome-wide empirical distribution of the RM statistic under null hypothesis. The phenotypes of ETEC F4ab/F4ac susceptibility were randomly shuffled 10,000 times; and the empirical critical value was determined by choosing the 95th percentile of the highest test statistic over the 10,000 permutation replicates. We declared a significant SNP at a genome-wide 0.05 significance level if its RM statistic value was larger than the empirical critical value.

## Results

**Figure 1:**
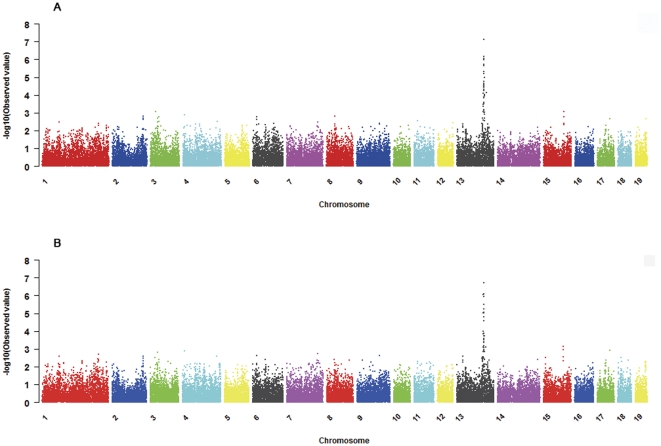
Manhattan plots of genome-wide association for ETEC F4ab/F4ac susceptibility. Negative log10-transformed *p* values of all tested SNPs for susceptibility to ETEC F4ab (Fig. 1A) and F4ac (Fig. 1B) are plotted against position on each of 19 chromosomes. Different chromosomes are represented by different colours. Chr. 19 stands for the X chromosome of swine.

The profiles of the *p* values (in terms of −log10 *p*) of all tested SNPs for susceptibility to ETEC F4ab/F4ac are shown in Figure 1. The genome-wide significant SNPs detected by the RM test for ETEC F4ab/F4ac susceptibility at the permutation-based critical level are presented in Table 4. In total, 28 and 18 genome-wide significant SNPs (*p*<0.05) were detected for susceptibility to ETEC F4ab and F4ac, respectively, and all of the 18 significant SNPs for F4ac are also significant for F4ab. Furthermore, based on the most recent porcine genome sequence assembly (Sscrofa9.2), 18 SNPs (15 of them are significant for both F4ab and F4ac) among the 28 significant SNPs are located within an interval of about 2.6 Mb on SSC13, while the positions of other 10 SNPs are not available (see Table 4).

**Table 4:** Genome-wide significant SNPs for ETEC F4ab and F4ac susceptibility.

SNP name	Chr.	Position (bp)^a^	Nearest gene^b^		*p* value^c^	
			Name	Distance (bp)	F4ab	F4ac
**M1GA0027009**	0	NA	NA	NA	1.32E-06	2.77E-05
**DIAS0003141**	0	NA	NA	NA	6.47E-06	NS
**ALGA0122702**	0	NA	NA	NA	7.01E-07	NS
**ALGA0106843**	0	NA	NA	NA	9.08E-07	NS
**DIAS0001226**	0	NA	NA	NA	1.05E-06	NS
**MARC0066682**	0	NA	NA	NA	1.95E-06	2.61E-05
**M1GA0027131**	0	NA	NA	NA	1.99E-06	4.55E-05
**DIAS0004305**	0	NA	NA	NA	9.22E-08	NS
**MARC0101456**	0	NA	NA	NA	2.22E-06	NS
**ALGA0109098**	0	NA	NA	NA	4.71E-06	NS
**MARC0095534**	13	100411276	*SENP5*	35104	6.47E-06	9.18E-06
**H3GA0037333**	13	100853976	*TNK2*	31111	3.25E-05	NS
**MARC0012378**	13	100878960	*TNK2*	6127	7.01E-07	8.97E-07
**M1GA0017682**	13	100885039	*TNK2*	48	9.08E-07	8.14E-07
**ASGA0058885**	13	100916770	*TNK2*	within	1.05E-06	1.11E-06
**MARC0067282**	13	101488856	*ZNF148*	within	1.95E-06	2.73E-05
**MARC0099692**	13	101550380	*ZNF148*	8634	1.99E-06	2.72E-05
**ALGA0072075**	13	101582070	*HEG1*	31668	7.22E-08	1.94E-07
**MARC0002946**	13	101604226	*HEG1*	9512	2.22E-06	9.87E-06
**ASGA0058925**	13	101659492	*HEG1*	within	4.71E-06	3.03E-06
**ASGA0089965**	13	101783439	*HEG1*	18479	1.77E-05	5.93E-06
**ASGA0091537**	13	101818006	*MUC13*	16416	2.32E-05	5.72E-06
**ALGA0106330**	13	101846502	*MUC13*	within	3.23E-05	8.97E-06
**H3GA0037348**	13	101925778	*ITGB5*	within	4.81E-05	1.64E-05
**H3GA0037351**	13	101955862	*ITGB5*	within	2.59E-05	9.06E-06
**MARC0096736**	13	102039909	*UMPS*	within	2.16E-05	NS
**DIAS0001297**	13	102070045	*UMPS*	21938	3.67E-05	NS
**H3GA0037388**	13	103041803	*MYLK*	144363	1.03E-05	2.68E-05

a Derived from the most recent porcine genome sequence assembly (Sscrofa9.2). NA: not available.

(http://www.ensembl.org/Sus_scrofa/Info/Index);

b The nearest known gene to the significant SNP;

c Obtained from the empirical distribution of test statistics via data permutation with 10,000 replicates. The thresholds for 5% (1%) genome-wise significance are 5.72E-05 (1.57E-06) for susceptibility to ETEC F4ab and 5.76E-05 (2.28E-06) for susceptibility to ETEC F4ac. NS: not significant.

**Figure 2:**
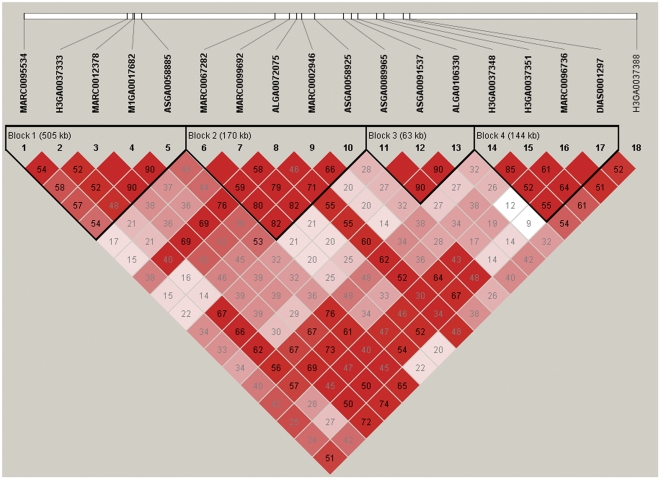
Linkage disequilibrium (LD) pattern for common significant SNPs on SSC13. LD blocks are marked with triangles. Values in boxes are LD (*r^2^*) between SNP pairs and the boxes are coloured according to the standard Haploview colour scheme: LOD>2 and *D′* = 1, red; LOD>2 and *D′*<1, shades of pink/red; LOD<2 and *D′* = 1, blue; LOD<2 and *D′*<1, white (LOD is the log of the likelihood odds ratio, a measure of confidence in the value of *D′*).

The LD patterns among the 18 significant SNPs with known positions are shown in Figure 2. Four LD blocks were defined with the criteria of Gabriel *et al.* [27]. Outside of these four blocks, there is merely one significant SNP (H3GA0037388) located about 972 kb away from its nearest significant SNP, which could be a long-distance LD marker, *i.e.*, it has strong LD with but a long physical distance from the causal mutation(s) of ETEC F4ab/F4ac susceptibility.

## Discussion

GWAS has been considered as a promising tool for gene identification for complex traits. So far GWAS in domestic animals are largely focused on economically important growth and production traits, such as milk production in dairy cattle, backfat in swine, *etc*. In this study, we carried out a GWAS to explore potential causal gene(s) for F4abR and F4acR in swine. To our knowledge, this is the first study aiming at unravelling the genetic mechanism of the ETEC F4ab/F4ac susceptibility in piglets using a case-control design based on a high density SNP chip panel. Findings from our study will lay a preliminary foundation for the follow-up functional validation of *F4abR/F4acR* candidate gene(s) in swine.

In the *vitro* adhesion test of our study, misclassification may occur for the weakly adhesive animals but seldom for strongly adhesive, adhesive and non-adhesive animals. Billey *et al.* [28] considered the weak adhesion was an artefact because it was rarely detected. This was the same case in our study, *i.e.*, the number of weakly adhesive piglets was merely 11 for F4ab, and 24 for F4ac. Python *et al.* suggested that the receptor for weak adhesion might be different from that for normal and strong adhesion [5], [6]. Hence it was reasonable to treat the weak adhesion as non-adhesion in contrast to the adhesion and strong adhesion. To further examine potential false-positive/false-negative errors raised from misclassification, we performed a GWAS using a subset of original samples without the weakly adhesive ones, and the association results (data unpresented) totally kept unchanged compared with those showed herein.

In the study, according to the critical value determined by permutation tests via 10,000 replicates, 18 genome-wide significant SNPs with known positions in porcine genome were identified for susceptibility to ETEC F4ab/F4ac. The positions of the 18 SNPs are consistent with previously reported QTL regions [3], [9]. These results offer a high possibility that *F4abR* and *F4acR* share the common causal mutation(s) as suggested by some previous studies [5], [7], [29]. Similar with the findings by Rampoldi *et al.* [10], no significant SNP was detected in the *MUC4-LMLN* region.

The LD patterns of the 18 significant SNPs for ETEC F4ab/F4ac showed that almost all of them are in high LD (*r^2^*) level even though there are long distances between some of them (see Figure 2). Further scrutinizing their positions, we found that they are located in either intergenic regions (n = 11) or intronic regions (n = 7) (see Table 4). Under the assumption that the causal mutation(s) are in strong LD with these significant SNPs, we are in effort to tag potential functional genes within the LD regions covered by these significant SNPs. This is also the common strategy adopted by a suite of prior similar studies [6], [30], [31]. The promising candidate genes were then determined in terms of their known functional information in other species. To further pinpoint the true causal mutation(s), follow-up functional validations should be performed by focusing on mining all possible mutations in coding or non-coding regions of these candidate genes.

Based on the most recent porcine genome sequence assembly (Sscrofa9.2, http://www.ensembl.org/Sus_scrofa/Info/Index), one SNP, ASGA0058885, is located within an intron of *TNK2* (tyrosine kinase non-receptor 2) and another two SNPs, MARC0012378 and M1GA0017682, are very close to *TNK2* (6127 bp and 48 bp away, respectively); Three SNPs, MARC0099692, ALGA0072075 (the most significant SNP) and MARC0002946, are located between *ZNF148* (zinc finger protein 148) and *HEG1* (HEG homolog 1); Two SNPs, ASGA0089965 and ASGA0091537, are located between *HEG1* and *MUC13* (mucin 13); Three SNPs, MARC0067282, ASGA0058925 and ALGA0106330, are located in introns of *ZNF148*, *HEG1* and *MUC13*, respectively; Two SNPs, H3GA0037348 and H3GA0037351, are located in introns of *ITGB5* (integrin beta-5). Based on these findings, we could further focus on these genes involved, *i.e.*, *TNK2*, *ZNF148*, *HEG1*, *MUC13* and *ITGB5*.

Among these five genes, the association of *TNK2* and *MUC13* with susceptibility to ETEC F4ab/F4ac were analysed in previous studies [30], [32]. *TNK2* encodes a tyrosine kinase that binds to CDC42 (cell division cycle 42 protein) in its GTP-bound form is inactivated by intrinsic hydrolysis of the nucleotide γ-phosphate, which can be stimulated by GTPase-activating proteins (GAPs) [33]. In humans, several alternatively spliced transcript variants have been identified from *TNK2*, and the full-length of two transcript variants has been determined. The homologue swine *TNK2* gene has a genomic length of about 42 kb and is located in the region of 100,885–100,927 kb on SSC13, which is very close to *MUC4*, so it makes *TNK2* to act as a possible candidate gene similar to *MUC4* based on its position as well as its functional aspect. However, according to the results of Joller [32], the sequence variants of both *MUC4* and *TNK2* were not completely linked to the phenotypes, and none of them was causative for susceptibility to ETEC F4ab/F4ac. A similar result was reported by Rampoldi *et al.* [10], who also suggested that the causal mutation(s) was downstream of the gene *LMLN* and might be located around the region containing the *MUC13* gene. Therefore, *TNK2* should not be considered as a candidate gene for F4abR/F4acR in further research.

Similar to *MUC4*, *MUC13* belongs to the family of secreted and cell surface glycoproteins expressed by ductal and glandular epithelial tissues [34] and plays a role in cell signalling. *MUC13* should be a highly possible candidate gene having the causal mutation(s) for susceptibility to ETEC F4ab/F4ac since *MUC4* was denied with quite reasonable experimental evidences [10]. Zhang *et al.* [30] showed that the expression pattern of the porcine *MUC13* mRNA in tissues was similar to humans, with highest level in jejunum and moderate levels in trachea, stomach and liver, and the SNPs detected in *MUC13* were strongly associated with susceptibility to ETEC F4ab/F4ac in a White Duroc×Erhualian resource population in their initial study. Subsequently, *MUC13* was assigned as a positional candidate gene for F4abR/F4acR via the study about a pig–human comparative radiation hybrid (RH) map [31]. However, no causal mutation can be identified in *MUC13* so far.

The other three genes, *ZNF148*, *HEG1* and *ITGB5*, have not been treated as candidate genes for F4abR/F4acR so far. *ZNF148* has been shown to be involved in regulation of T cell receptors in human [35], but no evidence is available to indicate its functional relationship with bacterial infection or molecular receptor on epithelial cells. We are strongly in favour of *HEG1* and *ITGB5* as potential candidate genes for F4abR/F4acR based the following reasons.

From the physical positions of these two genes, *MUC13* is flanked by *ITGB5* and *HEG1*, each transcribed from the forward strand. Interestingly, *HEG1* and *MUC13* were proved sharing same molecular features, suggesting they might be evolutionarily related [36]. However, the expression pattern of *HEG1* in human is quite different from that of *MUC13* based on the information from the BioGPS (http://biogps.org), which showed a low expression level for *HEG1* but the highest expression level for *MUC13* in small intestine. The functional information of this gene is limited so far, and it is not clear whether it is possible that *HEG1* has the causal mutation(s) responsible for the ETEC F4ab/F4ac susceptibility or not. In spite of these facts above, our study revealed three significant SNPs (including the most significant one) close to *HEG1* and one within it, which suggests that *HEG1* could be considered as a candidate gene and merit follow-up validation in the future.

Like *HEG1*, based on the same database, the expression level of *ITGB5* is lower than that of *MUC13* in small intestine of human, but it is not an essential issue because the expression pattern is not stable in different ages or species. And, some functional information of *ITGB5* proved in previous studies shows its potentiality of being *F4abR/F4acR* gene. The protein type of *ITGB5* belongs to the integrin beta chain family, and is associated with alpha-V for compounding integrin αVβ5, which plays an important role in the innate defence system against bacterial infection by influencing the rapid turnover and exfoliation of mucosal epithelial cells [37]. In human, αVβ5 is a major endocytic receptor for vitronectin (Vn) which has an Arg-Gly-Asp (RGD) sequence for binding [38], [39], [40]. Vn plays an important role in bacterial serum resistance, adhesion and internalization mediated by host cell signalling. Furthermore, it has distinct binding sites for pathogens and epithelial cells like a cross-link between bacteria and epithelial cells [41]. In addition, Vn bound to *E. coli, Staph. aureus and S. pneumoniae* provides a more efficient bacterial adhesion to epithelial cells [42]. Therefore, it is extremely possible that a potential mutation in porcine *ITGB5* could affect integrin αVβ5 binding to Vn, and as a result its accompanying *E. coli* could not adhere to jejunal epithelial cells. Moreover, it has been reported that the polymorphisms of *ITGB5* is the host factor which might affect adenovirus infection and decrease lung function in human [43]. Additionally, the *ITGB5* subunit was found on both the apical and basal surface of epithelial cells and its expression is essentially oestrous cycle-independent in mice [44]. Based on the evidences above and the results in our study, we confirm that *ITGB5* is the most possible functional candidate gene for the F4abR/F4acR.

In this study, individuals from three different swine breeds were involved in the association analysis, so the issue of population stratification should be a major concern which needed to be addressed. Since the RM test itself is immune to population stratification [45], [46], it is safe to assume that the SNPs detected have convincing associations with ETEC F4ab/F4ac susceptibility in swine with a reasonable false discovery rate.
